# Comparison and Validation of Ichthyoplankton DNA Extraction Methods

**DOI:** 10.3390/mps4040087

**Published:** 2021-12-06

**Authors:** Diouri Lamia, Uwiringiyeyezu Théophile, Abdelouahab Hinde, Malki Mohamed, Baibai Tarik, Soukri Abdelaziz

**Affiliations:** 1 Health and Environment Laboratory, Faculty of Sciences Ain Chock, University Hassan II, B.P 5366 Maarif, Casablanca 20000, Morocco; S-malki@hotmail.com; 2 National Institute of Halieutic Research, Street Sidi Abderrahmane Club Equestre Ould Jmel, Casablanca 20100, Morocco; hind.abdelouahab@gmail.com (A.H.); baibaitarik@gmail.com (B.T.); 3 Physiopathology, Molecular Biology and Biotechnology Laboratory, Faculty of Sciences Ain Chock, University Hassan II, B.P 5366 Maarif, Casablanca 20000, Morocco; tuwiringiyeyezu@yahoo.fr

**Keywords:** ichthyoplankton, identification, molecular tool, quantitative PCR

## Abstract

Ichthyoplankton is the cluster of planktonic organisms that consists of fish eggs and larvae. These planktonic stages belong to the temporary zooplankton, representing future exploitable stocks. The study of the early ontogenesis of fish plays a key role in the understanding and evaluation of these populations through the study of their abundance and their spatio-temporal distribution. To better understand and protect these fisheries resources, it is essential to identify the different stages of fish embryonic development. This identification is usually performed using the classical method, based on morphological criteria under a binocular magnifying glass; however, this methodology is not always sufficient and is time consuming and, therefore, it is necessary to rely increasingly on molecular tools. The major problem with these tools is the yield and quality of the nucleic acids extracted from ichthyoplankton, especially in the case of eggs, which are small. Several methods have been used for DNA extraction from ichthyoplankton, either automated or manual, but very often from larvae or adults. In the present work, five fish egg DNA extraction protocols were compared based on their DNA yield and extraction quality, verified by agarose gel electrophoresis and quantitative PCR amplification. The results showed that extraction by our heat-protocol for direct PCR (Hp-dPCR) presents the simplest and cheapest protocol of all the kits used in this study, providing a sufficient quantity and quality of nucleic acids to be used for PCR amplification, and being within the reach of third world laboratories that often do not have sufficiently large budgets to obtain automated kits.

## 1. Introduction

Fish eggs and larvae are part of the ichthyoplankton. These planktonic stages belong to the temporary zooplankton or meroplankton, representing future exploitable stocks [1]. The study of the early life stages of ichthyoplankton plays a major role in understanding the ecology and evolution of fish faunas and their constituent populations and is an important component in fisheries resource assessment models through the study of fish fauna abundance and spatio-temporal distribution [2]. To better understand and protect these fisheries resources, it is necessary to identify the different stages of fish embryonic development. The identification of ichthyoplankton is usually carried out based on morphological criteria under a binocular magnifying glass, according to such features as the size and shape of the eggs, the pigmentation of the embryo, the presence or absence of adipose drops, the yolk structure, and the size of the perivitelline space [2]. However, this method is not always sufficient and is time-consuming [3] and, therefore, it is necessary to make increasing use of molecular tools [4]. The major problem of this type of approach is the failure of amplification arising mainly from the extraction of nucleic acids from ichthyoplankton. This applies especially in the case of eggs that are of small size and reflects the small amount of genetic material, the situation becomes even more difficult when dealing with samples consisting of a single individual. The appropriate choice of DNA extraction technique is very important, including the sample fixation step, which plays an important role in the efficiency of the extraction of the genetic material [5].

Several methods have been used for the extraction of DNA from eggs, including the phenol/chloroform/isoamyl (PCI) method [6], the Chelex method [7], and automatic kits, such as those using the principle of magnetic beads which can be efficient but which are very expensive [8]. Therefore, it has become necessary to look for a protocol that is both efficient and less expensive. It is within this framework that the present study was carried out: five fish egg DNA extraction protocols were compared based on their DNA yield and their extraction quality verified using agarose gel electrophoresis and by quantitative PCR amplification.

**Experimental Design:**


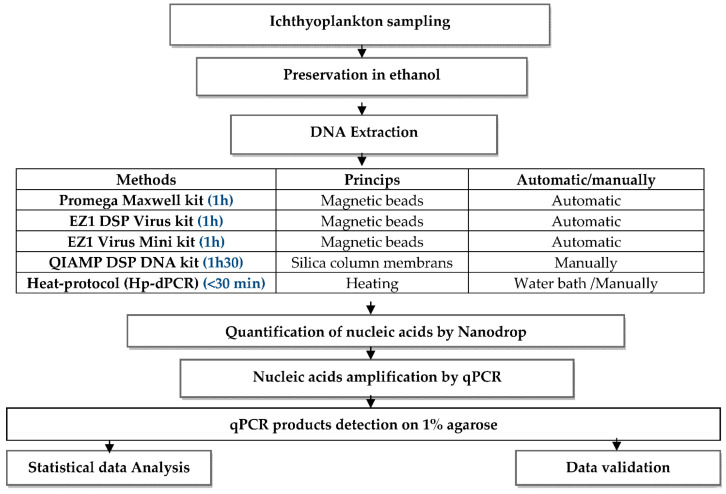


## 2. Materials and Methods

I. Ichthyoplankton sampling

Fish egg sampling was conducted during an oceanographic cruise aboard the research vessel N/R Al Amir Moulay Abdellah in the Moroccan Mediterranean in October 2019. The collection of ichthyoplankton eggs was carried out using a Bongo net of 20 cm diameter and 150 µm mesh size, at a maximum depth of 100m, by oblique strokes, according to the step method [9]. The samples were then preserved in ethanol. A total of 50 samples of the species *Spicara smaris* (*Linnaeus*, *1758*) (0.89 mm diameter) belonging to the family Centracanthidae were selected.

II. Extraction of nucleic acid
  1. Extraction with EZ1 DSP Virus Kit (Qiagen, Hilden, Germany) and EZ1 Virus Mini Kit (Qiagen, Hilden, Germany)

To an Eppendorf tube containing the egg we added 75 µL of sample reagent MTB/RIF (Cepheid, SE-171, 54 Solna, Sweden) and 15 µL of protease Q (Qiagen, Hilden, Germany). The nucleic acid extraction steps were performed automatically on the EZ Advanced XL (Qiagen, Hilden, Germany) according to the manufacturer’s instructions.

The protocol is available at: https://www.qiagen.com/us/products/diagnostics-and-clinicalresearch/solutions-for-laboratory-developed-tests/ez1-advanced-xl-instrument (accessed on 21 July 2021).

2. Extraction with the QIAamp DSP DNA Kit (Qiagen, Hilden, Germany)

To an Eppendorf tube containing the egg, 75 µL of sample reagent MTB/RIF buffer (Cepheid, Sunnyvale, CA, USA) and 15 µL of Q protease (Qiagen, Hilden, Germany) were added. The extraction steps with the QIAamp DSP DNA Kit (Qiagen, Hilden, Germany) were performed manually. A quantity of 90 µL of AL buffer was added, followed by vortexing carefully for 15 s, incubation at 70 °C for 10 min, followed by light centrifugation of the tube to remove the drops from the lid. Then 200 µL of ethanol (96–100%) was added, followed by vortexing for 15 s and light centrifugation of the tube to remove the drops from the lid. The mixture was pipetted onto the QIAamp mini-column, and then the continuation of the process was performed according to the manufacturer’s instructions

The protocol is available at: https://www.qiagen.com/ma/resources/resourcedetail?id=566f1cb1-4ffe-4225-a6de-6bd3261dc920&lang=en (accessed on 21 July 2021).

3. Extraction with the Promega Maxwell Kit (Promega Corporation, Madison, WI, USA)

This extraction was based on the use of magnetic beads with a Maxwell RSC automatic extractor (Promega, Madison, WI, USA). Using Promega kits, 100 µL of lysis buffer was added to each egg with 30 µL of proteinase K solution. The mixture was vortexed for 10 s. Then each tube of the heating block was incubated and set to 56 °C for 20 min. The continuation of the process was performed according to the manufacturer’s instructions.

The protocol is available at: https://worldwide.promega.com/products/nucleic-acid-extraction/genomic-dna/maxwell-16-tissue-dna-purification-kit/?catNum=AS1030 (accessed on 15 June 2021).

4. Heat-protocol for direct PCR (Hp-dPCR) (Our protocol)

This direct extraction technique did not require sophisticated machines or any special extraction kits. To a tube containing the egg, we added 90 µL of the buffer containing 10 mM Tris-HCl (pH 8.3), 20 mM KCl, 10% SDS, 20% Triton X100, and 0.05% Tween, and let incubate for 10 min. To the mixture was added 10 µL of proteinase K (Qiagen, Hilden, Germany). The mixture was then incubated in a water bath or block heater for 15 min at 56 °C, followed by inactivation for 10 min at 95 °C, and centrifugation for 1 min at maximum speed. Then, the extraction product was directly quantified by NanoDrop and real-time PCR amplification

III. Quantification of nucleic acids

After extraction, an assay with a NanoDrop Lite Spectrophotometer (Thermo Scientific, Madison, WI, USA) was performed to quantify the extracted DNA in ng/µL and to evaluate possible contamination (e.g., by proteins, polyphenols). The purity of the extract was determined by the ratio of the absorbance measured at 260 nm to the absorbance measured at 280 nm. The DNA was considered sufficiently pure when the R ratio was between 1.8 and 2. A value below 1.8 indicates contamination by proteins. Finally, the DNA extracts were stored in a freezer at −20 °C.

IV. Amplification of nucleic acids

Real-time PCR reactions were performed in duplicates, using FISH1[TCAACCAACCACAAAGACATTGGCAC] and FISH2[TCAACCAACCACAAAGACATTGGCAC]. Conditions of cycling were as follows: a preheating step at 94 °C for 5 min, 35 cycles of amplification (94 °C for 45 s, 55 °C for 45 s, 72 °C for 60 s) and a final extension step at 72 °C for 7 min.

The PCR reaction mixture of total volume 25 μL, contained 0.2× SYBR^®^ Green I (from 10,000 × stocks, Invitrogen, Carlsbad, CA, USA), 5 nM fluorescein (Invitrogen, Carlsbad, CA, USA), 50 mM KCl, 20 mM Tris, pH 8.4 at 25 °C, 0.1% Triton X-100, 2.5 mM MgCl_2_, 0.1% dimethyl sulfoxide, 0.2 μM of each primer, 0.2 mM dNTP, 1 U of Taq DNA polymerase, and 5 μL of the DNA sample. PCR products were detected on 1% agar gel in TBEX1 buffer added with 2.5 µL ethidium bromide.

V. Statistical analysis

The data obtained in our experiment were subjected to statistical study. The results were analysed by Grubbs’ Tests. https://www.graphpad.com/quickcalcs/Grubbs1.cfm (accessed on 1 September 2021).

## 3. Results

In this study, five automatic and manual extraction protocols applied to eggs were compared. Each isolated egg was extracted by each protocol. The purity and yield of the genomic DNA samples were quantified with NanoDrop in order to evaluate parameters, such as quality and quantity (Table 1).

DNA concentrations (ng/µL) and the 260/280 ratio were determined. The concentrations ranged from 0.8 ± 0.329 to 170 ± 0.717 (ng/µL) with a ratio ranging from 1.3 to 1.8. The highest level of DNA purity (A260/A280) was obtained by the heat-protocol for direct PCR (Hp-dPCR) (our protocol) in the presence of the modified lysis solution, followed by the Promega Maxwell Kit method, then the EZ1 Virus Mini Kit method, then after QIAamp, finally by the EZ1 DSP Virus Kit method. The highest yield was obtained with the modified Heat-protocol for direct PCR (Hp-dPCR) (our protocol) followed by the Maxwell Kit (170 ± 0.717 and 50 ± 0.053 ng/µL, respectively). The other protocols gave low yields and ratios lower than 1.8, the ratio accepted as pure DNA. The modified Heat-protocol (Hp-dPCR) was the fastest of the methods evaluated and was cheaper and less toxic. The other automated protocols were also effective, but were time-consuming and the DNA was diluted except for the Promega Maxwell Kit method which gave fairly good values, though lower than those obtained using our protocol (Hp-dPCR).

It is evident that our protocol, by virtue of its simplicity and especially its use of easy-to-prepare solutions, and which is also not overly expensive and does not require sophisticated equipment, constitutes a solution for laboratories with limited resources (see Table 2 below).

To confirm purity for use in the PCR and subsequent sequencing, we performed a real-time PCR following the protocol described in Section 2. Partial cytochrome c oxidase I (coI) gene sequences of 650 bp were amplified by PCR using primers FISH1 and FISH2 [10], the amplification efficiency for each protocol are summarized in Table 3.

Using the Ct threshold as a measure of efficiency of the tested protocol gave the following results: (Hp-dPCR > Promega Maxwell Kit > QIAMP DSP DNA Kit > EZ1 Virus Mini Kit (undetectable) and EZ1 DSP Virus Kit (undetectable). The results for the PCR products showed that the method of Heat-protocol for direct PCR (Hp-dPCR) (our protocol) was very reliable as the concentration of nucleic acids extracted by this method was an average of 170 ± 0.717 ng/µL which explains the Ct of 18.32 ± 0.7043 in PCR amplification. Reliable results were also obtained using the Promega Maxwell Kit (Ct: 20.54 ± 0.4403) and the QIAamp DSP ADN Kit (33.87 ± 0.746), but the EZ1 DSP Virus Kit and EZ1 Virus Mini Kit did not provide results that would warrant their use on eggs, especially since these kits were designed specifically for viruses.

**Figure 1:**
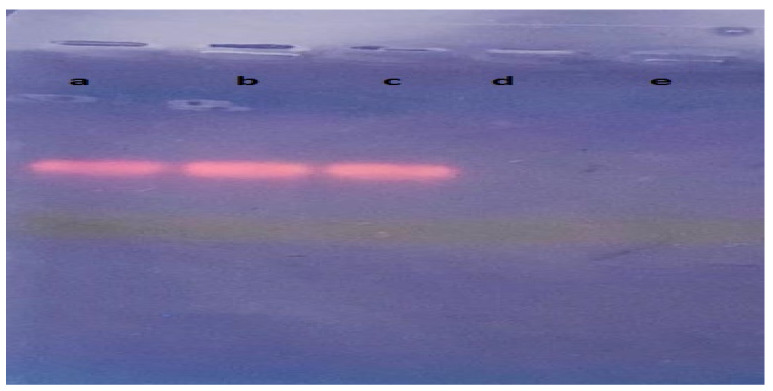
Agar gel electrophoresis. From left to right: amplification products for (**a**) Heat-protocol for direct PCR (Hp-dPCR) (our protocol); (**b**) Promega Maxwell Kit; (**c**) QIAamp DSP DNA Kit; (**d**) EZ1 Virus Mini Kit, and (**e**) EZ1 DSP Virus Kit.

In parallel, we checked the intensities of the amplification products on 1% agarose gel. The intensities of the PCR products were consistent with the result of the real-time PCR, with almost similar band intensities for the three protocols: Hp-dPCR, Promega Maxwell Kit, and QIAamp DSP DNA Kit. The other two protocols did not show any bands (Figure 1).

## 4. Discussion

The molecular identification of ichthyoplankton is a very important step in the assessment of ichthyoplankton stocks. Until now, the identification has been performed mainly using the larvae or adults. This allows evaluation of the impact of the environment on them. Commercial kits specifically prepared for extraction of nucleic acids from fish eggs are rare. Therefore, in this study, we compared different extraction methods to assess their reliability when used on fish eggs, including kits already available on the market, as well as our own heating method in the presence of optimized protease Q (Hp-dPCR). The identification of ichthyoplankton, and more particularly their eggs, has, for a long time, utilized the classical method of observation using a binocular magnifying glass. However, this process is very limited and many eggs remain unidentified hence the usefulness of molecular methods. The latter is dependent on the quantity and quality of the DNA to be extracted.

Several studies have reported methods using the principle of magnetic beads, such as the EZ1 DSP Virus Kit, the EZ1 Virus Mini Kit, and the Promega Maxwell Kit, used in this study. These automatic extraction kits are validated by the manufacturers and various studies have reported their effectiveness. However, in our study, we obtained diluted results with these protocols, which may be related to the fact that the kits are specific for bacterial and viral DNA. We may also have lost amounts of DNA in the pre-treatment step, but our objective was achieved, which was to validate the methods that can be used in such studies. The specific kits for eggs are not available in our region, so we decided to use those kits at our disposal according to the manufacturer’s instructions. These kits are adaptable to the types of samples and the purpose of extraction, such as purification of total RNA, mi RNA, poly A+ mRNA, DNA, or protein, by following the pre-treatment instructions.

Several methods have been described in the literature, including the Invitrogen kit used for DNA extraction from eggs of Cyprinidae species [11], the phenol/chloroform/isoamyl (PC1) method [6,8], and the CTAB method used in *Fundulus grandis* species [12] and *Aedes aegypti* species [13]. These methods have produced very different yields and have highlighted the difficulties associated with the extraction of DNA from fish eggs.

The manual method of the QIAamp DSP DNA Kit using silica membranes gave comparable results to the Chelex-100 resin (Bio-Rad Laboratories, Irvine, CA, USA) which is an ion-chelating resin which has been used on *Cynoglossus joyneri* eggs by Zhou X. et al. [14] and in *Theragra chalcogramma* [15]. In the case of the latter, the DNA extraction yield was too low to amplify the DNA.

Methods that combine extraction and PCR amplification are highly sought after because they facilitate research, such as the method using the DEP-25 DNA Extraction Kit that requires only 25 min to extract nucleic acids in small samples of fish of the families Calliphoridae and Sarcophagidae [16].

A multitude of other kits and extraction methods described in the literature are used for DNA extraction from small samples, such as the RED Extract-N-Amp TM Tissue PCR Kit (Sigma Aldrich, Louis, MO, USA) [4], and the protocol using TNES release buffer used for the identification of larvae in the regions of Upper Paraná and Sao Francisco [17]. In addition, methods include the Nucleo Spin Kit, the Nucleo Spin XS, and the Hot SHOT Kit [18] which uses an alkaline lysis buffer (0.2 mM EDTA disodium, 25 mM NaOH, pH 12) to crush the eggs. Nevertheless, most of these kits are primarily used for DNA extraction from larvae or adults.

The protocol (Hp-dPCR) used in this study enables rapid extraction by incubating each egg in a lysis buffer in the presence of proteinase K. The final extraction product can be used directly in PCR. This is a simple, rapid and scalable method with a yield comparable to those used by Gleason and Burton [19].

## 5. Conclusions

The extraction of DNA from fish eggs by our method (Hp-dPCR) presents the simplest and cheapest protocol of all the kits compared in this study, providing a sufficient quantity and quality of products to be used for PCR amplification and being within the reach of laboratories whose budgets do not extend to the ordering of automated kits

## Figures and Tables

**Table 1:** The concentrations and 260/280 ratio obtained for each of the protocols. Each of the values represents an average value of 10 extractions per protocol. Mean values of statistical data and standard deviation curves were calculated using Grubbs’ Tests, in 50 samples used with 10 extractions for each protocol. Statistically significant values *p* < 0.05.

Extraction Method	Concentration ng/µL	R 260/280
EZ1 DSP Virus Kit (Qiagen, Hilden, Germany)	0.8 ± 0.329	1.5 ± 0.086
EZ1 Virus Mini Kit (Qiagen, Hilden, Germany)	7 ± 0.243	1.3 ± 0.256
QIAmp DSP DNA Kit (Qiagen, Hilden, Germany)	3 ± 0.598	1.6 ± 0.092
Promega Maxwell Kit (Promega Corporation, Madison, WI, USA)	50 ± 0.053	1.7 ± 0.624
Heat-protocol for direct PCR (Hp-dPCR) (Our protocol)	170 ± 0.717	1.8 ± 0.044

**Table 2:** Comparison of costs, extraction duration and required instruments of the 5 methods.

Kits	Costs/Kit	Costs/Test	Extraction Duration	Required Instruments
EZ1 DSP Virus Kit	* 475.00$/48 tests	9.89$	1 h	EZ1 advanced XL
EZ1 Virus Kit	* 443.00$/48 tests	9.23$	1 h	EZ1 advanced XL
QIAamp DNA Kit	* 259.00$/50 tests	5.18$	1 h	QIAcube
Promega Maxwell Kit	* 750.00$/48test	15.63$	1 h 30 min	Maxwell^®^ RSC
Hp-dPCR Method	Not on the market 3.00$/test	3.00$	<30 min	Water bath

* Prices are indicated with local taxes.

**Table 3:** The Ct value of qPCR determined for each of the extractions: Each of the values represents an average value of 10 extractions per protocol. Mean values of statistical data and standard deviation.

Extraction Method	Ct (Cycle Threshold)
EZ1 DSP Virus Kit (Qiagen, Hilden, Germany)	-------
EZ1 Virus Mini Kit (Qiagen, Hilden, Germany)	-------
QIAamp DSP DNA Kit(Qiagen, Hilden, Germany)	33.87 ± 0.746
Promega Maxwell Kit (Promega Corporation, Madison, WI, USA)	20.54 ± 0.4403
Heat-protocol for direct PCR (Hp-dPCR) (Our protocol)	18.32 ± 0.7043
